# [Not Quite] The Ketogenic Diet in a Pill

**DOI:** 10.15844/pedneurbriefs-29-4-3

**Published:** 2015-04

**Authors:** Andrew J. Kim

**Affiliations:** 1 Division of Neurology, Ann & Robert H. Lurie Children's Hospital of Chicago, Chicago, IL; 2 Departments of Pediatrics and Neurology, Northwestern University Feinberg School of Medicine, Chicago, IL

**Keywords:** Lactate Dehydrogenase, Epilepsy, Stiripentol, Ketogenic Diet

## Abstract

Researchers at Okayama University, Japan showed lactate dehydrogenase (LDH) inhibition suppresses neuronal excitation in vitro, reduces EEG discharges and seizures in rodent models, and may provide a novel mechanism for anticonvulsant medications in human patients.

Researchers at Okayama University, Japan showed lactate dehydrogenase (LDH) inhibition suppresses neuronal excitation in vitro, reduces EEG discharges and seizures in rodent models, and may provide a novel mechanism for anticonvulsant medications in human patients. Using basal ganglia brain slices to investigate the mechanism of the ketogenic diet (KD), they found neurons became hyperpolarized not from the ketone bodies per se, but from the reduced glucose concentration that accompany the switch to ketone bodies-based cerebral metabolism. Then through a series of experiments, they pinpointed pyruvate and oxaloacetate as the specific molecules that affect the K-ATP channel and membrane potential: less glucose leads to less pyruvate and oxaloacetate, which opens the K-ATP channels and hyperpolarizes the neurons. In vitro they reproduced this ketone bodies-induced neuronal suppression by inhibiting LDH, the enzyme that reversibly converts lactate to pyruvate. The researchers also inhibited LDH in vivo to reduce seizures and EEG paroxysmal discharges in pilocarpine and kainate rodent models. They then screened 20 current anticonvulsants and found that stiripentol alone had significant LDH inhibitor action. Lastly, they showed that isosafrole, a stiripentol analog and a more potent LDH inhibitor, had anticonvulsant activity and was well tolerated in rodent models. [1]

COMMENTARY. The authors report two important new findings: (1) the neuronal inhibition associated with the KD correlate with reduced pyruvate and oxaloacetate levels, and (2) LDH inhibitors that lower pyruvate and oxaloacetate levels work as anticonvulsants in vitro and in vivo models. Despite this shared mechanism, KD and LDH inhibitors have an important difference: LDH inhibitors do not provide an alternative fuel to make up for the lost pyruvate and oxaloacetate. LDH inhibition, therefore, may be more akin to selective fasting than the ketogenic diet. It's easier to imagine using LDH inhibitors to enhance the action of the ketogenic diet than to replace it. Either way, LDH inhibitors now join 2-deoxy-D-glucose [2] as potential compounds that inhibit seizures by altering cerebral metabolism.

Many hypotheses have been proposed for the anticonvulsant mechanism of the ketogenic diet. They include changes in the water and electrolyte balance, changes in cerebral energy reserve, direct anticonvulsant effects of ketone bodies and fatty acids, and indirect action by increasing GABA/glutamate ratio [3–5]. The novel mechanism proposed in this paper integrates three appealing ideas in neuroscience: (1) astrocytes providing energy to neurons via the lactate shuttle, (2) K-ATP channel functioning as a molecular link between cerebral metabolism and electrical activity, and (3) basal ganglia (subthalamic nucleus and substantia nigra pars reticulata) playing a key role in the propagation and termination of seizures [6–8].
